# Efficiency of zinc in alleviating cadmium toxicity in hydroponically grown lettuce (*Lactuca sativa* L. cv. Ferdos)

**DOI:** 10.1186/s12870-024-05325-9

**Published:** 2024-07-09

**Authors:** Farhad Behtash, Trifeh Amini, Seyed Bahman Mousavi, Hanifeh Seyed Hajizadeh, Ozkan Kaya

**Affiliations:** 1 Department of Horticulture, Faculty of Agriculture, University of Maragheh, Maragheh, 55136-553 Iran; 2 Department of Soil sciences, Faculty of Agriculture, University of Maragheh, Maragheh, Iran; 3 Republic of Turkey Ministry of Agriculture and Forestry, Erzincan Horticultural Research Institute, Erzincan, 24060 Turkey; 4 Department of Plant Sciences, North Dakota State University, Fargo, ND 58102 USA

**Keywords:** Leafy greens, Heavy metals, Micronutrient, Stress physiology, Toxicity

## Abstract

**Background:**

A study on photosynthetic and enzyme activity changes and mineral content in lettuce under cadmium stress has been conducted in a greenhouse, utilizing the modulated effect of zinc (Zn) application in the nutrient solution on lettuce. Zn is a micronutrient that plays an essential role in various critical plant processes. Accordingly, three concentrations of Zn (0.022, 5, and 10 mg L^− 1^) were applied to hydroponically grown lettuce (*Lactuca sativa* L. cv. Ferdos) under three concentrations of Cd toxicity (0, 2.5, and 5 mg L^− 1^).

**Results:**

The results showed that along with increasing concentrations of zinc in the nutrient solution, growth traits such as plant performance, chlorophyll index (SPAD), minimum fluorescence (*F0*), leaf zinc content (Zn), leaf and root iron (Fe) content, manganese (Mn), copper (Cu), and cadmium increased as well. The maximum amounts of chlorophyll a (33.9 mg g^− 1^FW), chlorophyll b (17.3 mg g^− 1^FW), carotenoids (10.7 mg g^− 1^FW), maximum fluorescence (*Fm*) (7.1), and variable fluorescence (*Fv*) (3.47) were observed in the treatment with Zn without Cd. Along with an increase in Cd concentration in the nutrient solution, the maximum amounts of leaf proline (5.93 mmol g^− 1^FW), malondialdehyde (MDA) (0.96 μm g^− 1^FW), hydrogen peroxide (H_2_O_2_) (22.1 μm g^− 1^FW), and superoxide dismutase (SOD) (90.3 Unit mg^− 1^ protein) were recorded in lettuce treated with 5 mg L^− 1^ of Cd without Zn. Additionally, the maximum activity of leaf guaiacol peroxidase (6.46 Unit mg^− 1^ protein) was obtained with the application of Cd at a 5 mg L^− 1^ concentration.

**Conclusions:**

In general, an increase in Zn concentration in the nutrient solution decreased the absorption and toxicity of Cd in lettuce leaves, as demonstrated in most of the measured traits. These findings suggest that supplementing hydroponic nutrient solutions with zinc can mitigate the detrimental effects of cadmium toxicity on lettuce growth and physiological processes, offering a promising strategy to enhance crop productivity and food safety in cadmium-contaminated environments.

## Background

Soil pollution with heavy metals (HMs) like cadmium (Cd) has adversely affected most agricultural lands and irrigation waters. Cd has negative effects on plants and human health particularly due to being mobile, water-soluble [1] and toxic [2]. Cd toxicity happens through its accumulation in plant cells via its easy adsorption by plants, which in the next step, could seriously threaten human health similar to many chemical toxins [3]. Various important plant processes are disturbed by Cd leading to severe problems [1]. For instance, Cd causes Fe deficiency with opposing impacts on chlorophyll, thylakoid membranes and their related processes, Reactive Oxygen Species (ROS) generation and accumulation and damages to DNA, genes, protein, and membrane rupture. Cd induces oxidative stress due to ROS over-generation [1]. Several methods have been applied to reduce the effects of Cd toxicity, a prevalent environmental hazard in plants. Zinc (Zn) application has achieved promising results due to similar structures of Zn and Cd leading to parallel chemical behavior [3, 4].

Zinc (Zn) is a micronutrient with various essential roles in plant processes like involvement in cell division, preserving membrane integrity and structure, photosynthesis, carbon metabolism, stomatal activity, protein synthesis, tryptophan enzyme activities and structures and plant metabolism [5]. Zn is a part of lipids, proteins, and auxin structures, announcing its important role in nucleic acid metabolism and growth-related actions [6]. Likewise, Zn has critical roles in enzymatic activity and structure with regulatory effects [7]. Zn exists in the form of Zn^2+^ in the soil with great importance in plant defense against stress conditions, in the preferred dosage [8]. Most importantly, Zn decreases Cd’s negative effects on plants [9] since its absorption rate is higher than Cd [10] and due to chemical similarity [3]. Accordingly, Zn has an antagonistic effect with Cd [11]. Zn absorption rate is directly related to its concentration in the soil [12]. However, the low mobility of Zn results in its deficiency in plants [13], announcing the importance of Zn addition to the soil and nutrient solutions since its deficiency enhances Cd uptake by plants. In fact, Zn concentration in the soil could affect Cd uptake by plants. Interestingly, Cd: Zn ratio is more important in this regard [3] in which reduced Cd: Zn ratio via Zn application, as an appropriate agronomic practice, could mostly decrease Cd toxicity effects in areas with high Cd through lessening bioavailable Cd [14]. Previous studies reported positive effects of Zn addition on mitigating Cd toxicity on different plant processes [15, 16] including lettuce [3].

Lettuce (*Lactuca sativa* L.) is one of the most cultivated and consumed leafy greens, which is rich in many minerals (e.g. Fe, Mn, P, K), fiber, vitamins, phenolic compounds, flavonoids, and beta-carotene with benefits for human health [17]. Frequently, lettuce is hydroponically grown even under limited water supplies [18]. Additionally, Cd accumulation in lettuce could be considered as serious problem for human health [4] since lettuce could easily absorb HMs, leading to their accumulation in leaves that can cause harmful effects on humans. Therefore, Cd contamination of leafy greens is more important than other crops due to higher Cd accumulation in leaves [3, 4, 14]. All emphasize the importance of application of appropriate tactics like Zn application to reduce Cd content and toxicity impacts particularly considering little knowledge about lettuce response to Cd and applied treatments in this regard.

Considering the potential of Zn to alleviate Cd toxicity in plants, in addition to its beneficial roles as a micronutrient in various plant processes, the present study aimed to evaluate the application of zinc (Zn) through mineral solutions as a potential strategy to enhance the tolerance of lettuce (*Lactuca sativa* L.) against cadmium (Cd) toxicity. Our specific objectives were to assess the effects of Zn supplementation on several agronomic, physiological, and biochemical parameters of lettuce under Cd stress conditions. This comprehensive evaluation was undertaken to elucidate the potential of Zn supplementation as a viable approach to mitigate the deleterious effects of Cd stress on lettuce cultivation. The parameters analyzed aimed to quantify the impact of Zn supplementation on plant growth, biomass accumulation, and yield components under Cd stress. Physiological analyses were conducted to assess the effects on photosynthetic efficiency, water relations, and nutrient uptake, as these processes are often impaired by Cd toxicity. Additionally, biochemical assays were performed to evaluate the role of Zn in modulating antioxidant defense systems, enzymatic activities, and other metabolic pathways involved in stress tolerance mechanisms. This multifaceted approach was justified by the need to develop sustainable and efficient crop management strategies to counteract the detrimental consequences of Cd contamination in agricultural systems. Hence, by elucidating the potential of Zn supplementation in alleviating Cd toxicity in lettuce, this study aimed to contribute to the development of practical and environmentally friendly techniques for enhancing crop productivity and quality under adverse environmental conditions.

## Results and discussion

### Physiological traits

**Fig. 1:**
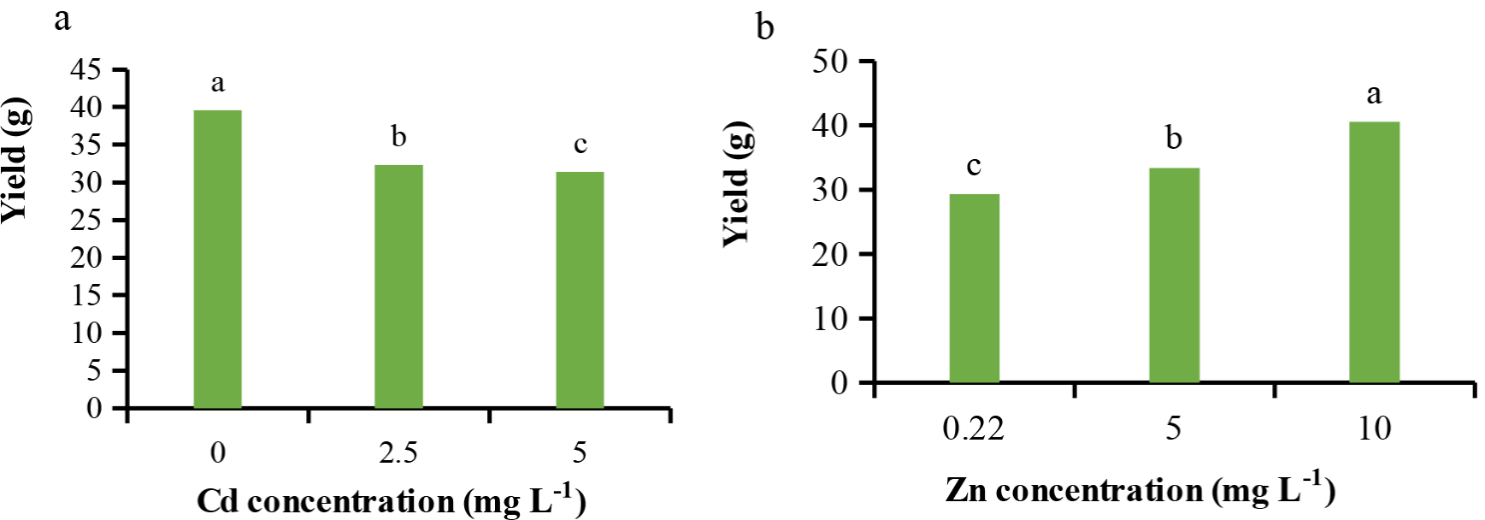
Effect of different concentrations of (**a**) Cd on (**b**) Zn on yield of *L. sativa* cv. Ferdos. Means not sharing the same letter do not differ significantly at *p* ≤ 0.01

As illustrated in Fig. 1a and b, Cd and Zn treatments resulted in a 26.11% decrease and a 38.22% increase in yield, respectively. The highest yield was obtained with the highest concentration of Zn. However, Zn application did not affect the yield under Cd toxicity conditions. We hypothesize that Cd toxicity reduced various agronomic traits [3, 19] due to decreased water and nutrient uptake and transport, photosynthesis, respiration [19], cell division, expansion and enlargement, and carbohydrate synthesis [20], which is corroborated by the present findings. The reduction in yield under Cd toxicity conditions has been previously reported in plants [21]. Fresh weight (FW) and dry weight (DW) of lettuce species were negatively impacted by Cd toxicity [4]. On the other hand, Zn application, as an essential micronutrient, enhanced agronomic traits, including FW, DW, and subsequently yield, due to increased water and nutrient uptake and transport, as well as induced cell division, enlargement, and hormonal metabolism [22], aligning with the current observations. Zn is known to play a role in auxin metabolism, conferring benefits to plant morphological and agronomic traits [6]. Additionally, Zn application (zinc sulfate) could potentially mitigate the detrimental effects of Cd on growth and other agronomic traits [23]. We hypothesize that Zn exerts critical functions in cell division, enlargement, photosynthesis, tryptophan and protein synthesis, and membrane integrity, all of which contribute to improved growth parameters, even under stress conditions [24]. Furthermore, Zn may reduce Cd translocation to the aerial parts of plants, thereby mitigating the destructive effects of Cd [25]. These findings suggest that the observed results may stem from assumptions that Cd toxicity impairs various physiological processes essential for plant growth and development, while Zn supplementation can counteract these detrimental effects by enhancing nutrient uptake, cell division and expansion, photosynthesis, and hormonal metabolism, as well as reducing Cd translocation to the aerial parts of the plant.

**Fig. 2:**
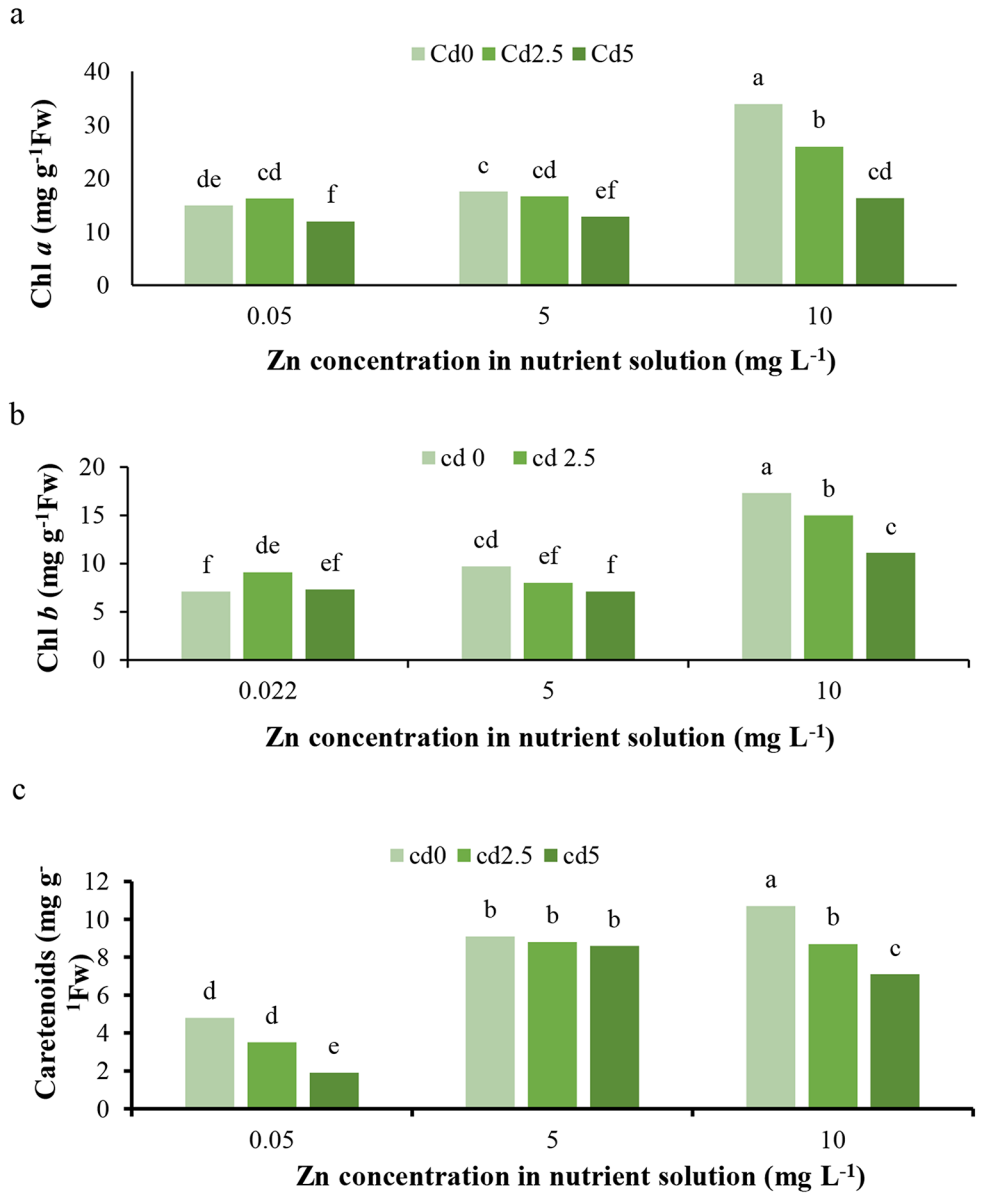
Interaction effect of different concentrations of Cd and Zn in nutrient solution on (**a**) Chl *a*, (**b**) Chl *b*, and (**c**) carotenoids of *L. sativa* cv. Ferdos leaves. Means not sharing the same letter do not differ significantly at *p* ≤ 0.01

**Fig. 3:**
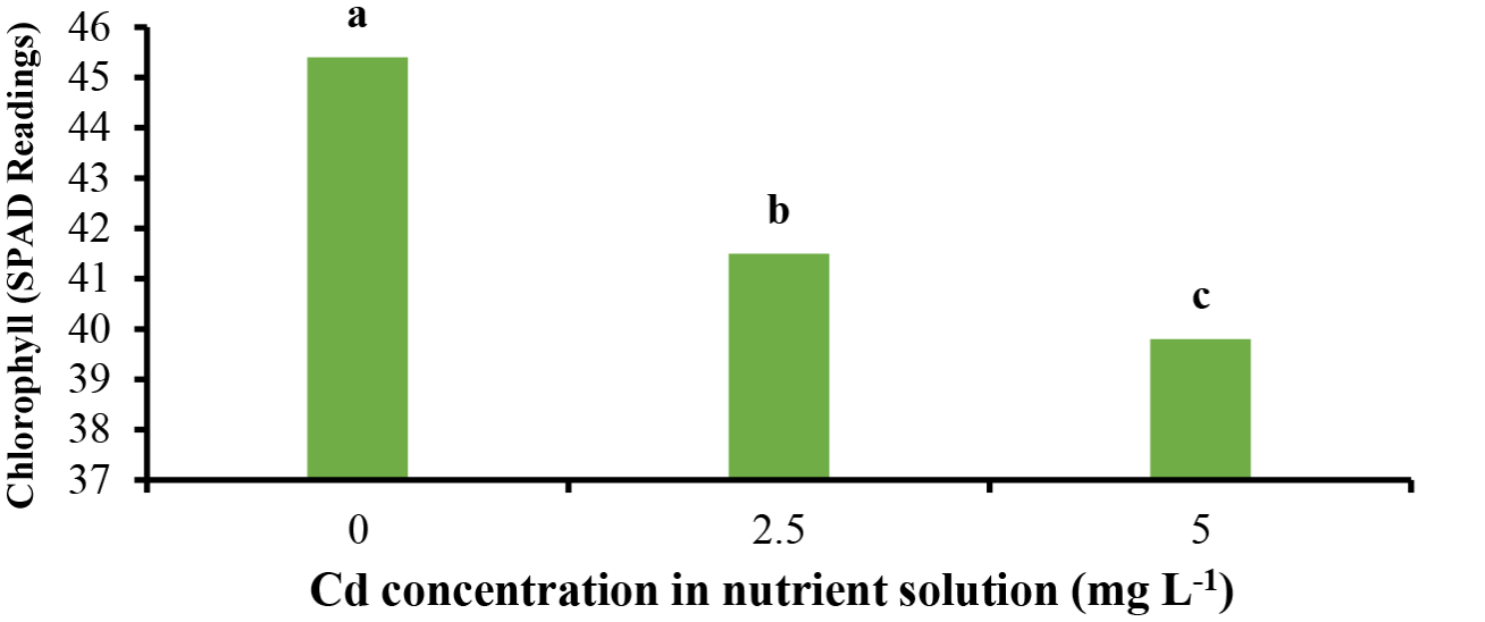
Effect of concentrations of Cd on chlorophyll of *L. sativa* cv. Ferdos. Means not sharing the same letter do not differ significantly at *p* ≤ 0.01

**Fig. 4:**
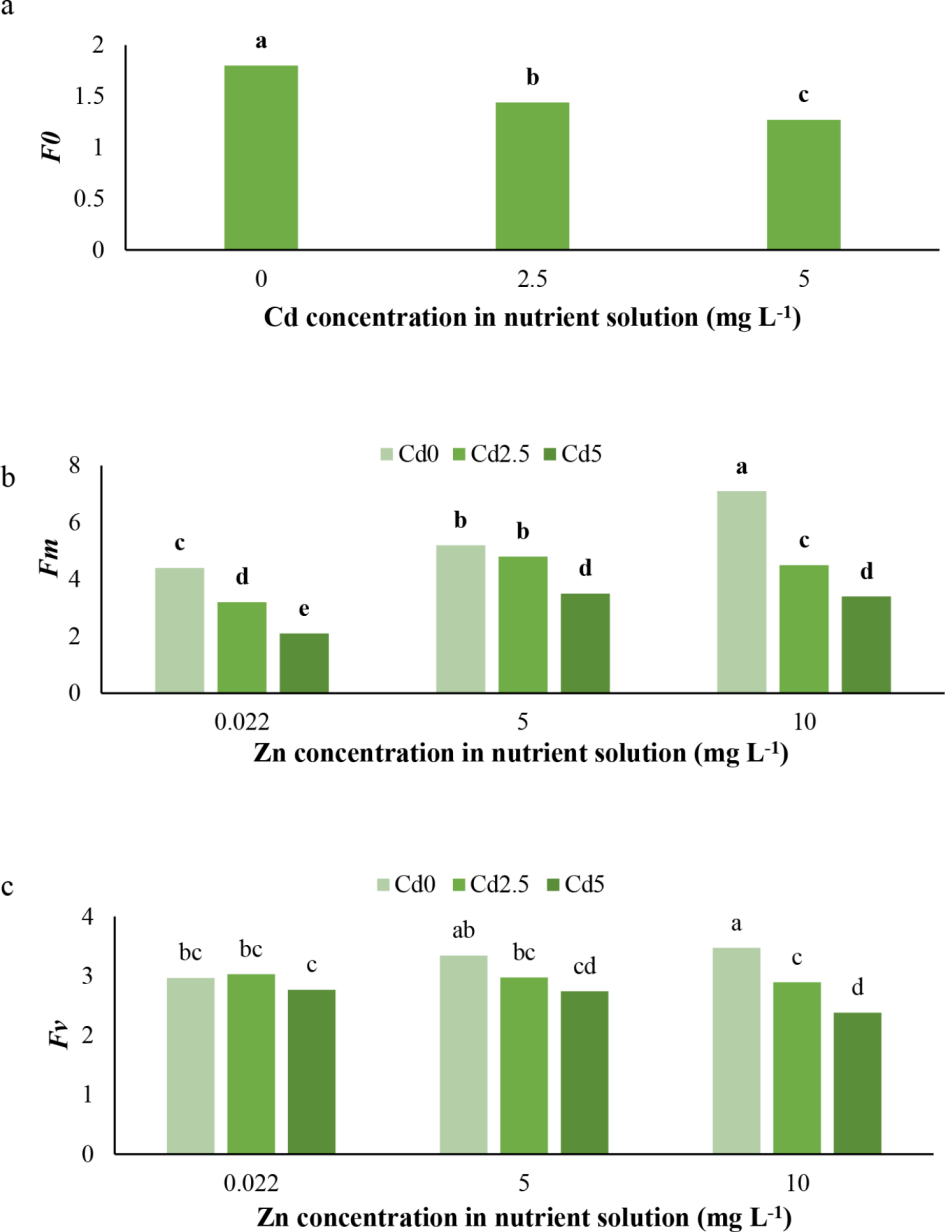
Effect of (**a**) different concentrations of Cd on *F0*, (**b**) interaction of different concentration on Cd and Zn on *Fm*, and (**c**) *Fv* value of *L. sativa* cv. Ferdos. Means not sharing the same letter do not differ significantly at *p* ≤ 0.01

The results indicate that Cd toxicity at 5 mg L^− 1^ reduced chlorophyll a (Chl *a*) content, while at 2.5 mg L^− 1^, it enhanced chlorophyll b (Chl *b*) (Fig. 2b) and reduced carotenoid content at both concentrations (Fig. 2c). Furthermore, Cd toxicity conditions decreased the SPAD (chlorophyll content index) (Fig. 3), minimum fluorescence (*F0*) (Fig. 4a), and maximum fluorescence (*Fm*) (Fig. 4b), while it had no effect on variable fluorescence (Fv) (Fig. 4c). In contrast, Chl *a* and *b* were positively affected by Zn applications under normal conditions. Under Cd toxicity conditions, Zn application at 10 mg L^− 1^ enhanced Chl *a* and *b* (Fig. 2a, b). Zinc applications increased carotenoid content under both normal and Cd toxicity conditions (Fig. 2c). Additionally, Zn applications enhanced SPAD under normal conditions but had no effect on SPAD under Cd toxicity (Fig. 3). The treatment with 10 mg L^− 1^ Zn enhanced *Fv* under normal and 5 mg L^− 1^ Cd toxicity conditions (Fig. 4c). *Fm* was positively affected by Zn treatments (Fig. 4b), whereas *F0* demonstrated no response to Zn application under either normal or Cd toxicity conditions. The Cd toxicity reduced Chl *a*, *b*, and carotenoids, as previously demonstrated [1, 20, 26], and chlorophyll fluorescence parameters, including *Fv/Fm*, Y(NO), and Y(II) [1, 19], by disrupting iron (Fe) absorption, which is necessary for chlorophyll and pigment synthesis and the photosynthesis process and apparatus [1, 2]. Additionally, Cd may induce damage to the photosynthetic apparatus, the light-harvesting complex, and photosystems I and II [27]. Furthermore, the Cd enhances the production of toxic ions and ROS, leading to the breakdown and reduction of photosynthetic pigments [1, 2]. Cadmium could potentially damage chloroplasts and thylakoid membranes, parallel to the damage to enzymes involved in chlorophyll biosynthesis, as well as activate enzymes involved in chlorophyll breakdown and ROS generation, all resulting in decreased chlorophyll synthesis and content [28]. The negative effects of Cd on chlorophyll content and photosynthesis in lettuce have been previously confirmed [28]. On the other hand, zinc is essential for chlorophyll biosynthesis, nitrogen (N) metabolism [29], carbon fixation and metabolism, and enzyme and protein biosynthesis and protection [30]. We claim that the enhancement in Zn increases N absorption, which plays a critical role in chlorophyll biosynthesis, presenting a secondary influence of Zn on chlorophyll content.

Additionally, Zn may provide the preservation of chlorophyll precursors, leading to chlorophyll biosynthesis [31]. Zinc application, at the preferred dosage, has been shown to enhance chlorophyll, carotenoids, and subsequently, photosynthesis [28]. Furthermore, Zn has been reported to increase higher stomatal conductivity and photosynthesis [32]. Zinc deficiency has resulted in negative impacts on photosynthetic pigments and photosynthesis due to a reduction in the activity of the carbonic anhydrase enzyme, indicating the necessity of Zn for chlorophyll biosynthesis [32]. Positive effects of Zn on chlorophyll biosynthesis and content in plants [29] and lettuce [33] under normal conditions have been previously recorded. Zinc application has been found to enhance chlorophyll biosynthesis and photosynthesis of hydroponically grown rice under Cd toxicity [15], possibly by enhancing protein synthesis, leading to chlorophyll biosynthesis and improved photosynthesis and chlorophyll fluorescence. Additionally, Zn may decrease Cd uptake and transfer, leading to a reduction in the toxic effects of Cd on photosynthetic pigments, apparatus, and activity [28].

**Fig. 5:**
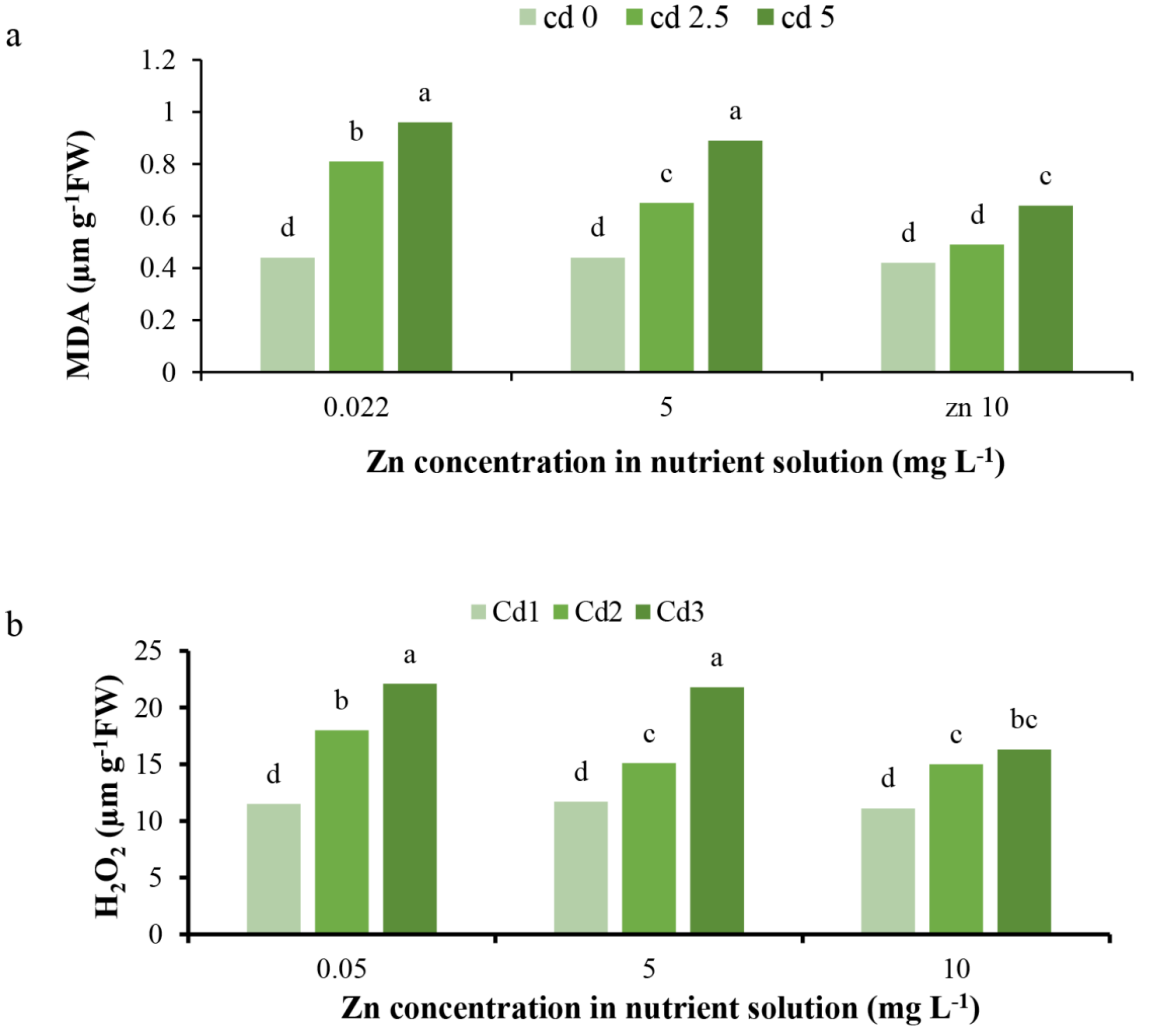
Interaction effect of different concentrations of Cd and Zn in nutrient solution on (**a**) MDA, and (**b**) H_2_O_2_ content of *L. sativa* cv. Ferdos leaves. Means not sharing the same letter do not differ significantly at *p* ≤ 0.01

The results demonstrate that Cd toxicity increased MDA and H_2_O_2_ values. Zn treatments had no effect on MDA and H_2_O_2_ values under normal conditions. However, under 2.5 mg L^− 1^ Cd toxicity, both Zn treatments reduced the content of MDA and H_2_O_2_, while under 5 mg L^− 1^ toxicity, only the 10 mg L^− 1^ Zn treatment decreased their values (Fig. 5a, b). The elevated MDA values indicate cell membrane damage and lipid peroxidation [34]. Similarly, higher H_2_O_2_ levels can cause damage to biological membranes and disrupt physiological processes by inducing oxidative stress [1]. Cadmium toxicity decreases membrane integrity through membrane damage, resulting in higher MDA levels [1], as previously confirmed [1, 34]. Specifically, Cd has been shown to increase MDA content in lettuce [34]. Additionally, the Cd toxicity leads to H_2_O_2_ accumulation by transferring electrons to oxygen instead of photosynthesis and respiration receptors [1], as well as through the interaction of Cd with antioxidant molecules [34], aligning with the current findings. On the other hand, Zn could bind to ROS and play a functional protective role against their damage to membrane lipids and proteins, thereby enhancing membrane integrity and decreasing potassium (K^+^) efflux [35]. Zinc is essential in maintaining membrane integrity, macromolecule (e.g., proteins, lipids) structure and protection, and nucleic acid metabolism [6]. Furthermore, Zn may activate antioxidant enzymes, detoxify ROS, and subsequently reduce the toxic effects of Cd and decrease H_2_O_2_ levels [26]. These findings suggest that Cd toxicity induces oxidative stress by increasing MDA and H_2_O_2_ levels, potentially due to membrane damage and disruption of electron transport chains, respectively. However, Zn supplementation may mitigate these effects by enhancing membrane integrity, activating antioxidant systems, and reducing ROS levels, thereby alleviating the toxic effects of Cd.

**Fig. 6:**
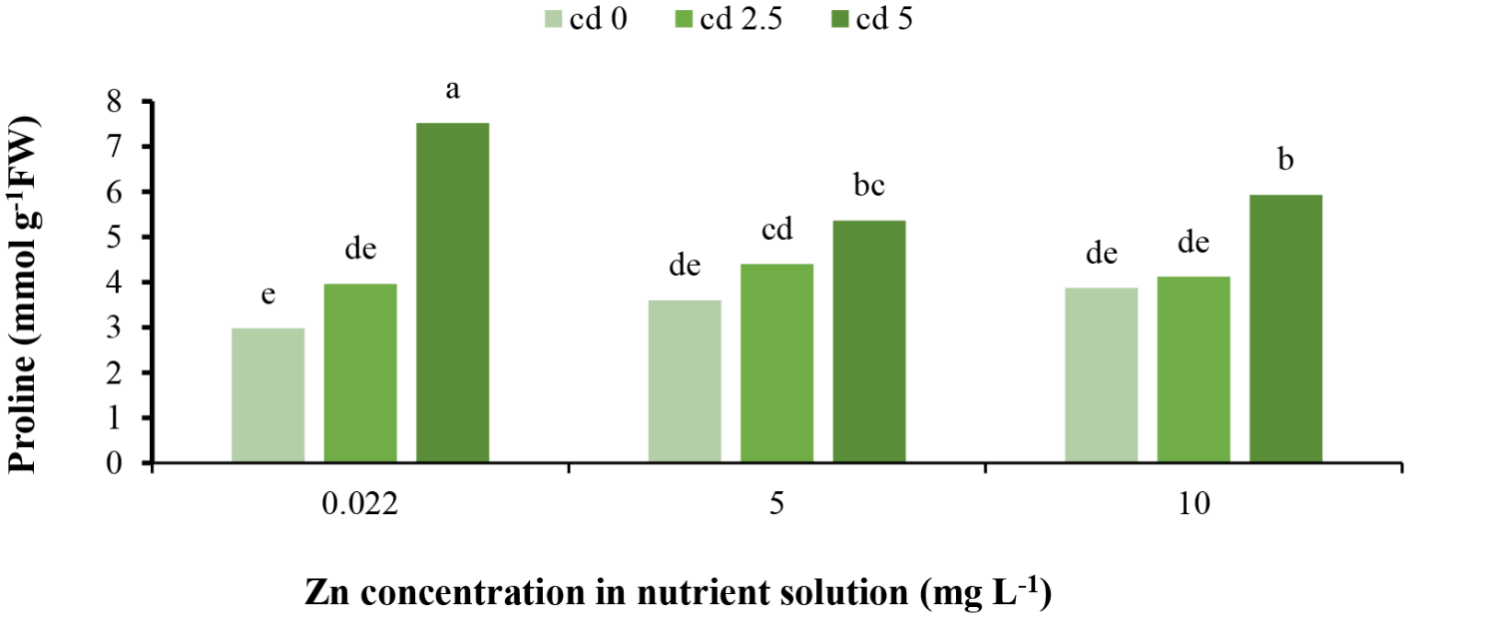
Interaction effect of different concentrations of Cd and Zn in nutrient solution on proline content of *L. sativa* cv. Ferdos leaves. Means not sharing the same letter do not differ significantly at *p* ≤ 0.01

The results indicate that proline content increased under 5 mg L^− 1^ Cd toxicity. Zinc applications did not impact proline content under normal conditions. None of the Zn treatments affected proline content in lettuce under 2.5 mg L^− 1^ Cd toxicity. However, under 5 mg L^− 1^ Cd toxicity, Zn applications reduced proline content (Fig. 6). It has been reported that under stress conditions, proline content increases to modulate the osmotic pressure of cells, preserve protein integrity, and interact with metal ions, thereby enhancing plant resistance, suggesting that proline acts as an antioxidant osmolyte and a molecular chaperone [1, 36]. Additionally, proline improves the metal-detoxification capacity of intracellular antioxidant enzymes and subsequently detoxifies ROS induced by stress conditions. Under heavy metal (HM) toxicity conditions, proline may cause higher antioxidant enzyme activities, improve cellular redox homeostasis, reconstruct chlorophyll, and regulate intracellular pH, thus acting as a metal chelator and protein stabilizer. The increase in proline content depends on the HM concentration, toxicity threshold, plant organ, and metal type [37], as observed for the 5 mg L^− 1^ Cd toxicity treatment. Proline enhancement has been previously reported under Cd toxicity conditions [1] in lettuce [38] to adjust osmotic pressure, stabilize macromolecule and organelle structures, and serve as a major reservoir of energy and nitrogen. On the other hand, Zn application causes an increase in chlorophyll and leaf surface area, leading to enhanced photosynthesis and proline content [39]. Furthermore, the Zn application has been shown to enhance proline content under Cd toxicity conditions [19]. This suggests that Cd toxicity induces proline accumulation as a defense mechanism against oxidative stress, osmotic imbalance, and macromolecule destabilization. However, Zn supplementation may reduce the need for proline accumulation by alleviating the toxic effects of Cd, potentially through improved photosynthesis, antioxidant activity, and metal chelation.

**Fig. 7:**
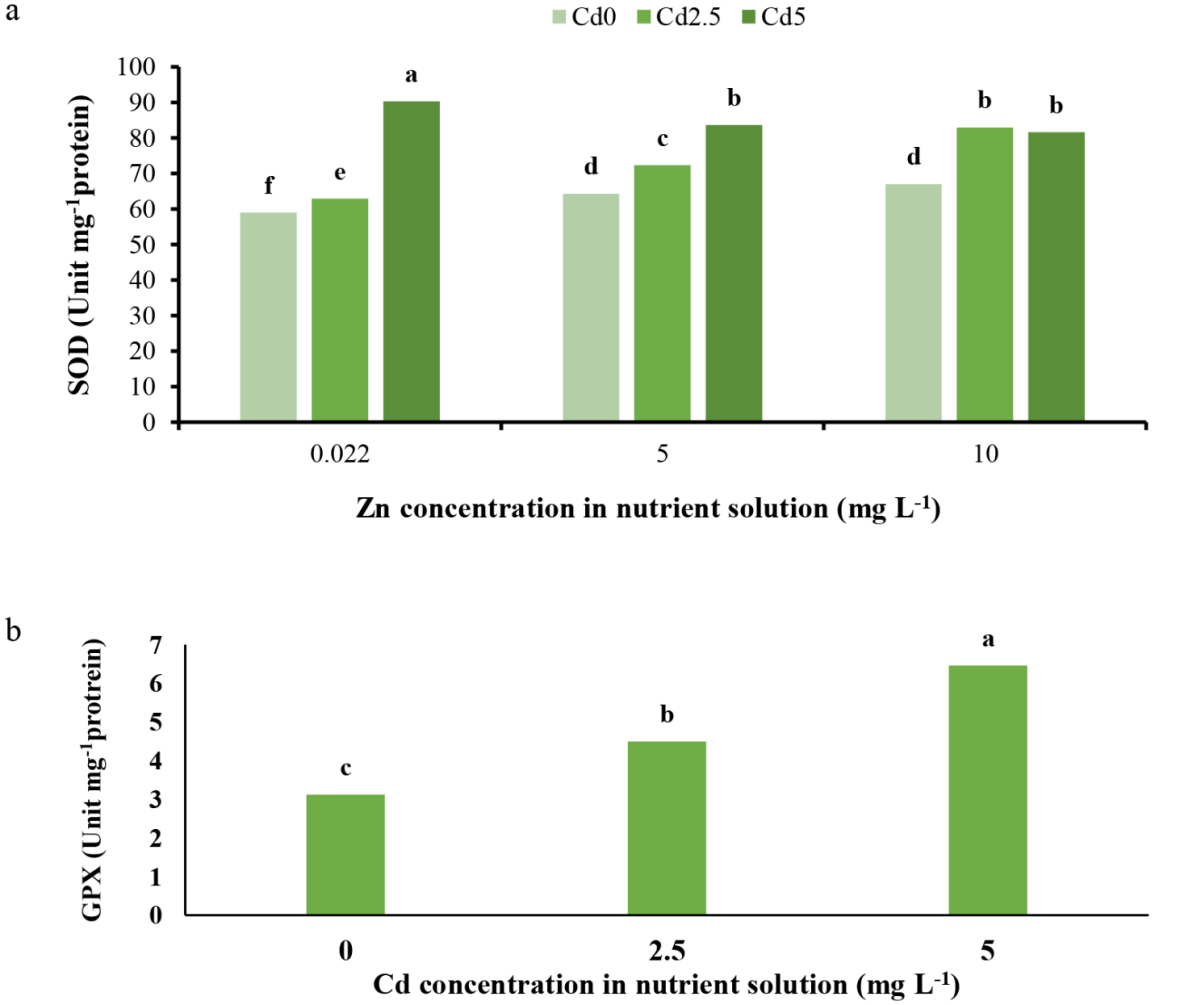
(**a**) Interaction effect of different concentrations of Cd and Zn in nutrient solution on SOD activity and (**b**) effect of different concentrations of Cd on GPX activity of *L. sativa* cv. Ferdos leaves. Means not sharing the same letter do not differ significantly at *p* ≤ 0.01

The results demonstrate that SOD and GPX enzyme activities increased under Cd toxicity conditions (Fig. 7a and b). Zinc treatments increased SOD activity under normal and 2.5 mg L^− 1^ Cd toxicity conditions, whereas the treatments decreased SOD activity under 5 mg L^− 1^ Cd toxicity (Fig. 7a). However, GPX activity was not affected by Zn applications under either normal or Cd toxicity conditions (Fig. 7b). It has been reported that SOD and GPX, as antioxidant enzymes and part of the antioxidant system, neutralize Cd-induced oxidative stress due to the overgeneration of ROS [34]. Furthermore, we postulate that SOD and GPX could protect cells from oxidant damage by alleviating the adverse effects of ROS through modulating ROS production and destruction [1]. SOD activity has been shown to enhance under Cd toxicity [34, 40], and Cd toxicity has been reported to cause an increase in SOD and GPX activities [1], most likely via enhanced activity of the glutathione-ascorbate cycle [40], aligning with the current findings. On the other hand, Zn plays roles in enzymatic catalysis and preserving enzyme structures [41]. Zinc enhances antioxidant enzyme activities to decrease the damaging impacts of ROS and free radicals [42]. Additionally, the Zn is a part of the SOD enzyme [43] and plays essential roles in the gene expression involved in ROS scavenging [42]. Zinc application has been shown to enhance SOD activity under Cd toxicity [43]. Under stress conditions, Zn application may cause higher antioxidant enzyme activities, and Zn application has been reported to enhance protein content in plants and activate antioxidant enzymes [40]. However, in some cases, SOD enzyme activity could decrease under Zn application and Cd toxicity due to higher Cd toxicity. Furthermore, SOD and GPX activities have been observed to decrease under Zn deficiency [42]. This suggests that Cd toxicity induces oxidative stress, leading to an increase in the activities of antioxidant enzymes such as SOD and GPX as a defense mechanism. Zinc supplementation may further enhance SOD activity under normal and moderate Cd toxicity conditions, possibly through its role in enzyme catalysis, structure, and gene expression related to ROS scavenging. However, under severe Cd toxicity, Zn application may reduce SOD activity due to the overwhelming effects of Cd toxicity. Additionally, Zn does not appear to significantly influence GPX activity in this study.

The results indicate that Cd content in leaves and roots increased under Cd toxicity conditions, as expected, the higher the level of Cd toxicity, the higher the Cd content. The Zn application had no effect on Cd content in leaves and roots under normal conditions. However, the Zn application at both concentrations (5 and 10 mg L^− 1^) reduced Cd content in leaves and roots under Cd toxicity conditions (2.5 and 5 mg L^− 1^), with the 5 mg L^− 1^ Zn treatment being the most effective (Table 1). According to Table 1, we conclude that Zn content in roots and leaves was negatively affected by Cd toxicity conditions. Conversely, Zn application enhanced Zn content in leaves and roots under normal conditions. The higher Zn concentration, the higher the Zn content. The Zn applications (5 and 10 mg L^− 1^) resulted in higher Zn content in lettuce leaves under both Cd toxicity conditions; however, its applications had no effect on Zn content in root tissues under the toxicity conditions. Furthermore, the results demonstrate that Cd toxicity reduced Fe content in roots and leaves. The Zn application increased Fe content in roots; an increase in Zn concentration resulted in a significant increase in Fe content under normal conditions. Under 2.5 mg L^− 1^ Cd toxicity, both Zn concentrations resulted in increased Fe content in roots, whereas under 5 mg L^− 1^ Cd toxicity, Zn applications decreased Fe content. The Zn application at 5 mg L^− 1^ enhanced Fe content in leaves, while at 10 mg L^− 1^, it had no effect on the content under normal and 2.5 mg L^− 1^ Cd toxicity conditions. However, Zn applications decreased Fe content in leaves under the 5 mg L^− 1^ Cd toxicity condition. Additionally, the results show that Mn content in roots was reduced under Cd toxicity conditions. A similar reduction was reported by Zn application under normal and mostly Cd toxicity conditions. We assert that Cd toxicity at 2.5 and 5 mg L^− 1^ concentrations enhanced and reduced Mn content in leaves, respectively. In contrast to the roots, we think that Zn application enhanced Mn content in leaves under normal and both Cd toxicity conditions. We hypothesize that higher Cd content in leaves and roots could be expected under Cd toxicity conditions due to its mobility property, competition for absorption and transferring sites, and easy transferring [2], as previously established [1, 40] in lettuce [4]. We assume that Cd toxicity resulted in Cd enhancement and Zn reduction [44]. Especially, leafy greens can chelate heavy metals (HMs) like Cd and decrease their absorption and transfer [45]. Soil and nutrient solution pH, the content of Mn, Fe, and Zn, Cd: Zn ratio, temperature, Cd content, and plant species could affect Cd uptake and transferring [3, 4]. We think that since Cd has similar chemical behavior to Zn, the decrease in Cd content by Zn application could be explained by this property, leading to the uptake and transport of Zn instead of Cd [44]. The Zn and Cd uptakes could occur via the same routes due to their antagonistic behavior and their negative action toward each other [11]. Furthermore, we claim that Zn has a higher absorption rate than Cd, leading to its preference by plants in the presence of both Cd and Zn [10]. Additionally, Zn uptake enhances in the case of its abundance in the soil or nutrient solution. It has been noted that Cd toxicity decreased Zn content due to Cd-induced changes in the molecules connected to Zn [10] and competition for Mn^2+^, Fe^2+^, and Zn^2+^ receptors and transporters [3]. Conversely, Zn application decreased Cd content in roots and leaves [46]. In fact, Zn application decreases Cd uptake and transport due to chemical similarity [26]. The Zn application could decrease Cd content, toxicity effects, and its accumulation in plant tissues [16, 20, 23, 25, 46]. In lettuce, we assert that Cd toxicity conditions enhanced roots and leaves Cd content, while Zn application decreased their Cd content [3]. Likewise, we think that Zn application enhanced Zn content in leaves and roots. The Cd exists in the Cd^2+^ form and, therefore, has close competition with Mn^2+^, Fe^2+^, and Zn^2+,^ resulting in their deficiency [47] via uptake by Zn, Mn, and Fe receptors [48]. An increase in Mn, Fe, and Zn content in soil and nutrient solutions could reverse this process and decrease Cd while enhancing Mn, Fe, and Zn, as observed in the current study to some extent [16]. The Cd toxicity causes an imbalance in nutrient uptake due to anatomical and structural changes made by Cd in roots and shoots [47]. The Cd toxicity resulted in a reduction in Fe, Mn, Mg, and Ca [2]. Additionally, Cd decreased Fe^2+^ uptake in lettuce roots [16]. Similarly, Cd toxicity led to a decrease in Zn and Mn in lettuce [2]. These observations could be explained by Cd competition for the absorption sites of nutrients with chemically similar behavior [49].

**Table 1:** Interaction effects of different concentrations of cd (Cd1 = 0, Cd2 = 0.5, and Cd3 = 5 mg ^L−1^) and zn (Zn1 = 0.022, Zn2 = 5, and Zn3 = 10 mg L^− 1^) in nutrient solution on Zn, Cd, Fe, Mn, and Cu content in Leaf and root of *L. Sativa* Cv. Ferdos leaves

Different Treatments	Zn Concentration (mg kg^− 1^)	Cd Concentration (mg kg^− 1^)		Fe Concentration (mg kg^− 1^)		Mn Concentration (mg kg^− 1^)		Cu Concentration (mg kg^− 1^)	
	Leaf	Leaf	Root	Leaf	Root	Leaf	Root	Leaf	Root
Zn1Cd1	47.7f	5.3f	1.9f	207.6ef	1043.7 h	14.1e	51.2e	7.3c	1.9 g
Zn1Cd2	37.8 g	33.9d	31.9d	234.4d	1101.3 g	20.1d	31.4c	11.6bc	24.2e
Zn1cd3	25.6 h	89.9a	78a	261.7c	1390.1a	9.1 g	31.1c	11.9b	41.6c
Zn2cd1	69.5c	5.6f	6.8e	413.5a	1287.6c	23c	41.5b	10.2d	7f
Zn2cd2	66.2c	44.53e	26.6d	295.3b	1251.7d	24.4b	32.9c	10.7 cd	26.6e
Zn2cd3	53.5e	66.6b	52.4b	196.9 fg	1214.2e	12f	8.7e	11.3bc	52.4b
Zn3cd1	92.9a	5.6f	9.7e	222.4de	1323.6b	30.6a	33.9c	10.8 cd	9.7f
Zn3cd2	77.2b	22.8e	15e	241.9 cd	1265.5c	36.4b	21.1d	11.6bc	31.6d
Zn3cd3	62.7d	55e	41c	186.6 g	1169.4f	15.93e	13e	12.8a	59a

Means not sharing the same letter do not differ significantly at *p* ≤ 0.01

## Conclusion

The present study provided valuable insights into the modulating effects of Zn application on Cd toxicity in hydroponically grown lettuce (*Lactuca sativa* L.). The findings demonstrate that Cd toxicity adversely affected various agronomic, physiological, and biochemical parameters in lettuce, including reduced growth traits, chlorophyll content, photosynthetic efficiency, and nutrient uptake, as well as increased oxidative stress, lipid peroxidation, and antioxidant enzyme activities. However, Zn supplementation through nutrient solutions effectively mitigated the detrimental impacts of Cd toxicity on lettuce plants. Zinc application also enhanced growth parameters, photosynthetic pigments, and chlorophyll fluorescence, while reducing the accumulation of ROS and associated oxidative damage. Moreover, Zn treatments decreased Cd uptake and translocation to aerial plant parts, thereby alleviating the toxic effects of Cd. For the scientific community and industry stakeholders, the study provided insights into the underlying mechanisms by which Zn mitigates Cd toxicity, such as enhancing antioxidant systems, membrane integrity, and nutrient homeostasis. Study also differs from existing research by providing a comprehensive evaluation of the role of Zn in alleviating Cd toxicity in lettuce, a widely consumed leafy green vegetable. Unlike many previous studies that focused on model plants or cereal crops, this research specifically investigated the response of lettuce, which is crucial for understanding the implications of Cd contamination and potential mitigation strategies for leafy greens consumed by humans. Additionally, the study employed a hydroponic system, allowing for precise control of nutrient concentrations and minimizing the influence of various soil factors, thereby providing insights into the direct effects of Zn supplementation on Cd toxicity alleviation mechanisms. Further investigations could explore the molecular and genetic basis of these mechanisms, potentially leading to the development of more effective strategies for managing heavy metal stress in crop plants. From both an industrial and agricultural perspective, the results highlighted the potential of Zn supplementation as a practical and sustainable approach to mitigate Cd toxicity in leafy greens, particularly in areas with elevated Cd levels in soil or irrigation water. By reducing Cd accumulation in edible plant parts, this strategy could contribute to improving food safety and quality, addressing a critical concern for consumers and regulatory bodies. In conclusion, the findings lay the foundation for future research efforts aimed at developing sustainable and effective approaches for enhancing crop resilience and productivity in the face of environmental challenges.

## Materials and methods

### Plant materials, applied treatments and stress conditions

The seeds (Pakan Bazr Company, Isfahan, Iran) of lettuce (*Lactuca sativa* L.) cv. Ferdos were sterilized (sodium hypochlorite (NaOCl, 1% (v/v), 5 min), washed with distilled water for three times and lastly soaked in distilled water (15 min). Then, five seeds were planted into each 12-kg pot containing medium grain sand and watered with tap water every other day. After seedling emergence, planted pots were irrigated with half-strength Hoagland solution (pH:6.6, EC:1.55; Coolang et al. [50]) as illustrated in Table 2. Two weeks later, the pots, containing two strong seedlings, were irrigated with full-strength Hoagland solution and again after two more weeks zinc sulfate as zinc (Zn) source at 0.022, 5 and 10 mg L^− 1^ concentrations each in five replications; cadmium sulfate (as cadmium (Cd) source at 0, 2.5 and 5 mg L^− 1^ concentrations each in three replications; as the stress conditions were applied through full-strength Hoagland solution that continued up to the harvest. Control plants were irrigated in the same manner (first tap water, then half-strength Hoagland solution and finally full-strength Hoagland solution) until the harvest and received any Zn treatments and Cd stress conditions. All measurements were performed at the harvest stage (six weeks after the applications) with three replications for each assay of the parameters. The research greenhouse (24 − 18°C; 65–75% RH) of the Faculty of Agriculture, University of Maragheh, Maragheh, Iran (longitude 46°16’ E, latitude 37°23’ N, altitude 1485 m) was considered as the experimental site of the study using a CRD (completely randomized design) and factorial experiment with five replications.

**Table 2:** Composition and concentration of salts in the modified Hoagland solution of Coolong et al. [50]

Nutrients	Concentration (mg L^− 1^)	Nutrients	Concentration (g L^− 1^)
H_3_BO_3_	2.86	Ca (NO_3_)_2_.2H_2_O	0.47
MnCl_2_.4H_2_O	1.81	KNO_3_	0.3
ZnSO_4_.7H_2_O	0.22	MgSO_4_. 7H_2_O	0.25
Na_2_MOO_4_.2H_2_O	0.02	NH_4_H_2_PO_4_	0.06
CuSO_4_.5H_2_O	0.08	FeEDTA	0.1

## Leaf fresh and dry weights and yield

Leaf fresh (FW) and dry (DW) weights were assessed through first weighing one randomly selected plant’s leaf which was then placed in the oven (70 ◦C, 72 h) for DW measurement. The yield was achieved through weighing aerial parts of all plants of each treatment.

### Leaf fresh and dry weights and yield**Photosynthetic pigments (Chl*a***, **Chl*b*and carotenoids), SPAD and chlorophyll fluorescence parameters (*Fv***, ***Fm***, **and*F0*)**

The absorbances at 645 nm (for Chl *b*), 663 nm (for Chl *a*), and 470 nm (for carotenoids) were measured using a spectrophotometer (UV-1800 Shimadzu, Japan) on the supernatants of acetone (3% v/v) extracted from the leaves. These absorbance values were then converted into precise amounts [51]. Leaf chlorophyll concentrations, indicated by SPAD values, were determined using a SPAD-meter (502 Plus Chlorophyll Meter, Japan) [52]. Chlorophyll fluorescence parameters, including Fv, Fm, and F0 values, were recorded using a dual-pam-100 chlorophyll fluorometer (Heinz Walz, Effeltrich, Germany) [53].

## Malondialdehyde (MDA)

For MDA determination, leaf samples (0.1 g) were homogenized in acetic acid (2.5 mL; 10% w/v), and then thiobarbituric acid (0.5% w/v) in trichloroacetic acid (TCA) (20%) was added to the obtained supernatants. The mixture was then incubated at 96 °C for 30 min. After incubation, the mixtures were cooled at 0 °C for 5 min, followed by centrifugation (10,000 rpm, 5 min). The absorbance of the resulting solution was recorded at 532 nm and 600 nm using a spectrophotometer and converted to MDA content [54].

### Hydrogen peroxide (H_2_O_2_)

For H_2_O_2_ measurement, the supernatant (0.5 mL), obtained from leaf samples (0.5 g) digested with trichloroacetic acid (5 mL, 0.1% w/v) in an ice bath, was mixed with potassium phosphate buffer (0.5 mL, pH 6.8, 10 mM) and potassium iodide (2 mL, 1 M). This mixture was then incubated in the dark for 30 min, and the absorbance was recorded at 390 nm. A standard calibration curve, previously prepared using various H_2_O_2_ concentrations, was used to calculate the H_2_O_2_ content [55].

## Proline

Leaves (5 g) were digested using sulfosalicylic acid (10 mL, 3% w/v). Following centrifugation (1000 rpm, 4 °C), the resulting supernatant (2 mL) was combined with ninhydrin acid (2 mL) and glacial acetic acid (2 mL). This mixture was then incubated at 100 ºC for 1 h and subsequently cooled in an ice bath. After cooling, toluene (4 mL) was vigorously mixed with the solution for 20 s. The absorbance was measured at 520 nm using a spectrophotometer and then converted to precise proline values using a standard curve obtained with L-proline [56].

### Superoxide dismutase (SOD) and guaiacol peroxidase (GPX) enzymes activities

The supernatants obtained from the extraction of leaf samples (0.5 g) with potassium phosphate buffer (3 mL) containing 1% PVP (polyvinylpyrrolidone) (pH 7, 50 mM, 4 °C) were utilized to assess the activities of superoxide dismutase (SOD) and guaiacol peroxidase (GPX) enzymes. The protocols for enzyme activity assessment were adopted from the methods outlined by Li et al. [57].

### Leaf and root cd contents

After washing leaves and root samples with deionized water, they were placed in oven (65 °C, 48 h), powdered, digested with HNO_3_/HClO_4_ at 100 °C and lastly kept in furnace (550 °C, 5 h) to attain their ash. After cooling down and dissolving the ashes with HCl (10 mL, 2 N), Whatman filter paper (No.42) was used for filtering them to a volumetric flask (50 mL). Distilled water was added to achieve the final 50 mL volume. Cd content was recorded using atomic absorption spectrometer (Model CTA 3000, ChemTech, UK) [1].

### Leaf and root zn, Mn, Fe, and Cu Contents

Minerals concentration was measured by wet digestion [58]. The leaf and root samples were washed with deionized water and air-dried. Then, the leaves were dried in an oven at 550 ◦C for 6 h. After cooling to room temperature, 10 mL of a 65% HNO_3_ was added to the inorganic residue in the crucibles, and they were placed in the digester without heating for 1 day. The next day, the samples was heated at 65 ◦C for 3 h and then at 110 ◦C for 3 h. The final clear solutions were filtered with Whatman paper N.42 and were transferred to a 100 mL volumetric flask and volume was made up with deionized water. Fe, Zn, Mn, and Cu were determined directly in final digests using an atomic absorption spectrophotometry (UV-1800, Shimadzu, Japan).

### Statistics

The factorial experiment was conducted following a completely randomized block design with three replications. Data for the parameters were subjected to statistical analysis using MSTAT-C ver 2.1 software. Mean values were separated using the Duncan test at the levels of five and one% error probability. In line with the objectives of this study, a p-value threshold of less than 0.05 was established as the criterion for statistical significance.

## Data Availability

Correspondence and requests for materials should be addressed to H.S.H.
